# Sensors and Communications for the Social Good

**DOI:** 10.3390/s23052448

**Published:** 2023-02-22

**Authors:** Claudio Palazzi, Ombretta Gaggi, Pietro Manzoni

**Affiliations:** 1 Department of Mathematics, University of Padua, Via Trieste, 35131 Padova, Italy; 2 Department of Computer Engineering (DISCA), Universitat Politècnica de València, 46022 Valencia, Spain

This topical collection focuses on applying sensors and communications technologies for social good. Social good is typically defined as an action that provides some sort of benefit to the general public. In this case, the Internet connection, education, and health care are all excellent examples of social good. However, the development of new media and the explosion of online communities have added a new meaning to the term. Social good is now about global citizens uniting to unlock the potential of individuals, technology, and collaboration to create a positive societal impact.

This reprint includes the recent advances and novel contributions from academic researchers and industry practitioners in this growing area. Each article was assigned and reviewed by at least three experts in the field during the review process, with a rigorous multi-round review process. Consequent to the great support and dedicated work of numerous reviewers, we accepted 10 excellent articles covering various topics under the umbrella of “Sensors and Communications for the Social Good”. We will introduce these articles and highlight their main contributions in what follows.

The first paper focuses on how the need to provide safe environments and reduce the risks of virus exposure plays a crucial role in our daily lives. Contact tracing is a well-established and widely used approach to tracking and suppressing the spread of viruses. Most digital contact tracing systems can detect direct face-to-face contact based on estimated proximity without quantifying the exposed virus concentration. In particular, they rarely allow for the quantitative analysis of indirect environmental exposure due to virus survival time in the air and constant airborne transmission. The authors of [1] proposed an indoor spatiotemporal contact awareness framework (iSTCA), which explicitly considers the self-containing quantitative contact analytics approach with spatiotemporal information to provide accurate awareness of the quanta concentration of virus in different origins at various times. Smartphone-based pedestrian dead reckoning (PDR) was employed to precisely detect the locations and trajectories for distance estimation and time assessment without additional infrastructure. The PDR technique calibrates the accumulative error by automatically identifying spatial landmarks. They utilized a custom deep-learning model composed of bidirectional long short-term memory (Bi-LSTM) and multi-head convolutional neural networks (CNNs) to extract the local correlation and long-term dependency to recognize landmarks. By considering the spatial distance and time difference in an integrated manner, they quantified the virus quanta concentration in the indoor environment at any time with all contributed virus particles. They conducted an extensive experiment based on practical scenarios to evaluate the performance of the proposed system. They showed that the average positioning error is reduced to less than 0.7 m.

Big Tech companies operating in a data-driven economy offer services that rely on their users’ data and usually store this personal information in “data silos”, which prevent transparency about their use and opportunities for data sharing for the public interest. In [2], the authors presented a solution that promotes the development of decentralized personal data marketplaces, exploiting the use of distributed ledger technologies (DLTs), decentralized file storages (DFSs), and smart contracts to store personal data and manage access control in a decentralized way. Moreover, they focused on the lack of efficient decentralized mechanisms in DLTs and DFSs for querying a specific data type. For this reason, the authors proposed using a hypercube-structured distributed hash table (DHT) on top of DLTs, organized for the efficient processing of multiple keyword-based queries on the ledger data. They tested their approach by implementing a use case for creating citizen-generated data based on direct participation and decentralized autonomous organization (DAO) participation. Performance evaluation demonstrated the viability of their approach for decentralized data searches, distributed authorization mechanisms, and smart contract exploitation.

In [3], the authors focused on how population aging requires innovative solutions to increase the quality of life and preserve autonomous and independent living at home. A need of particular significance is the identification of behavioral drifts. A relevant behavioral drift concerns sociality: Older people tend to isolate themselves. There is a need to find methodologies to identify if, when, and for how long the person is in the company of other people (possibly also considering the number). The challenge is to address this task in poorly sensorized apartments, with non-intrusive sensors that are typically wireless and can only provide local and simple information. The proposed method addresses technological issues, such as PIR (passive infrared) blind times, topological issues, such as sensor interference due to the inability to separate detection areas, and algorithmic issues. A house was modeled as a graph to constrain the transitions between adjacent rooms. Each room was associated with a set of values for each identified person. These values would decay over time and represent the probability that each person is still in the room. Because the used sensors cannot determine the number of people, this approach is based on a multi-branch inference that, over time, differentiates the movements in the apartment and estimates the number of people.

Sensor technology that captures information from the user’s neck region can enable a variety of new possibilities, including less intrusive mobile software interfaces. In [4], the authors investigated the feasibility of using a single inexpensive flex sensor mounted at the neck to capture information about head gestures, mouth movements, and the presence of audible speech. Different sensor sizes and various sensor positions on the neck were experimentally evaluated. With data collected from experiments carried out on the finalized prototype, a classification accuracy of 91% was achieved to differentiate common head gestures, a classification accuracy of 63% was achieved to differentiate mouth movements, and a classification accuracy of 83% was achieved in speech detection.

The work in [5] highlighted the fact that the linguistic and social impact of multi-culturalism cannot be ignored in any sector, creating the urgent need to create systems and procedures for managing and sharing cultural heritage in both supranational and multi-literate contexts. Text sensing is one of the most crucial research areas to achieve this goal. The long-term objective of the DigitalMaktaba project, born from an interdisciplinary collaboration between computer scientists, historians, librarians, engineers, and linguists, is to establish procedures for creating, managing, and cataloging archival heritage in non-Latin alphabets. In this paper, the authors discussed the currently ongoing design of an innovative workflow and tool in the area of text sensing, for the automatic extraction of knowledge and cataloging of documents written in non-Latin languages (Arabic, Persian, and Azerbaijani). The current prototype leverages different OCR, text processing, and information extraction techniques to provide highly accurate extracted text and rich metadata content (including automatically identified cataloging metadata), overcoming the typical limitations of current state-of-the-art approaches. The initial tests revealed promising results. The paper included a discussion of future steps (e.g., AI-based techniques further leveraging the extracted data/metadata and making the system learn from user feedback) and of the many foreseen advantages of this research, both from a technical and a broader cultural preservation and sharing points of view.

In [6], the authors focused on several smart home architecture implementations proposed in the last decade. These architectures are mostly deployed in laboratories or inside real habitations built for research purposes to enable ambient intelligence using various sensors, actuators, and machine learning algorithms. However, the major issues for most related smart home architectures are their price, proprietary hardware requirements, and the need for highly specialized personnel to deploy such systems. To address these challenges, lighter forms of smart home architectures known as smart homes in a box (SHiB) have been proposed. While SHiB remains an encouraging first step towards lightweight yet affordable solutions, they still suffer a few drawbacks. Indeed, some of these kits lack hardware support for some technologies, and others do not include enough sensors and actuators to cover the requirements of most smart homes. Thus, this paper introduced the LIARA Portable Smart Home Kit (LIPSHOK), designed to provide an affordable SHiB solution that anyone can install in an existing home. Moreover, LIPSHOK is a generic kit that includes four specialized sensor modules that have been independently introduced since the authors’ laboratory has been working on their development over the last few years. This paper first summarized these modules and their respective benefits within a smart home context. Then, it mainly focused on introducing the LIPSHOK architecture, which provides a framework to unify the use of the proposed sensors owing to a common modular infrastructure capable of managing heterogeneous technologies. Finally, the authors compared their work to existing SHiB kit solutions and revealed that it offers a more affordable, extensible, and scalable solution with resources distributed under an open-source license.

The ever-increasing pace of IoT deployment is opening the door to the concrete implementations of smart city applications, enabling the large-scale sensing and modeling of (near) real-time digital replicas of physical processes and environments. This digital replica could serve as the basis of a decision support system, providing insight into the possible optimizations of resources in a smart city scenario. In [7], the authors discussed an extension of prior work, presenting a detailed proof-of-concept implementation of a digital twin solution for the urban facility management (UFM) process. The Interactive Planning Platform for Adaptive Maintenance Operations for the City District (IPPODAMO) is a distributed geographical system fed and ingested heterogeneous data sources from different urban data providers. The data are subject to continuous refinements and algorithmic processes used to quantify and build synthetic indexes measuring the activity level inside an area of interest. IPPODAMO considers the potential interference from other stakeholders in the urban environment, enabling informed operations scheduling to minimize interference and operating costs.

The authors of [8] revealed that future university campuses will be characterized by a series of novel services enabled by the vision of the Internet of Things, such as smart parking and smart libraries. In this paper, the authors proposed a complete solution for a smart waste management system to increase the recycling rate on campus and better manage the entire waste cycle. The system is based on a prototype of a smart waste bin, which can accurately classify pieces of trash typically produced on campus premises with a hybrid sensor/image classification algorithm and automatically separate the different waste materials. The authors discussed the system prototype’s entire design, from the analysis of the requirements to the implementation details, and evaluated its performance in different scenarios. Finally, they discussed advanced application functionalities built around the smart waste bin, such as optimized maintenance scheduling.

Automation plays an important role in modern transportation and handling systems, e.g., controlling aircraft and ground service equipment routes in airport aprons, automated guided vehicles in port terminals or public transportation, handling robots in automated factories, drones in warehouse picking operations, etc. Information technology provides hardware and software (e.g., collision detection sensors, routing, and collision avoidance logic) that contribute to safe and efficient operations, with relevant social benefits in terms of improved system performance and reduced accident rates. In this context, the authors of [9] addressed the design of efficient collision-free routes in a minimum-sized routing network. They considered a grid and a set of vehicles, each moving from the bottom of the origin column to the top of the destination column. Smooth nonstop paths are required, without collisions or deviations from shortest paths, and they investigated the minimum number of horizontal lanes allowing for such routing. The problem is known as the fleet’s quickest routing problem on grids. They proposed a mathematical formulation solved for small instances using standard solvers. For larger instances, they devised heuristics that define priorities based on known combinatorial properties and design collision-free routes. Their experiments on random instances showed that their algorithms could quickly provide good-quality solutions.

Finally, in [10], the authors considered how the pandemic crisis has forced the development of teaching and evaluation activities exclusively online. In this context, the emergency remote teaching (ERT) process, which raised many problems for institutions, teachers, and students, led the authors to consider it important to design a model to evaluate teaching and evaluation processes. The study objective presented in this paper was to develop a model for the evaluation system called the learning analytics and evaluation model (LAEM). The authors also validated a software instrument they designed called the EvalMathI system, which is to be used in the evaluation system and was developed and tested during the pandemic. The evaluation process was optimized by including and integrating the dashboard model in a responsive panel. The EvalMathI dashboard monitored six online courses in the 2019/2020 and 2020/2021 academic years. For each of the six monitored courses, the curricula were evaluated through the analyzed parameters by highlighting the percentage achieved by each course on various components, such as content, adaptability, skills, and involvement. In addition, after collecting the data through interview guides, the authors determined the extent to which online education during the COVID-19 pandemic has influenced the educational process. Through the developed model, the authors also found software tools to solve problems raised by teaching and evaluation in the ERT environment.

The editors and authors express their thanks to the publisher and staff members for their ongoing dedication and valuable advice and encouragement, which helped to improve the quality of this reprint.
